# The Association between US Adolescents’ Weight Status, Weight Perception, Weight Satisfaction, and Their Physical Activity and Dietary Behaviors

**DOI:** 10.3390/ijerph15091931

**Published:** 2018-09-05

**Authors:** Furong Xu, Steven A. Cohen, Mary L. Greaney, Geoffrey W. Greene

**Affiliations:** 1 Department of Kinesiology, University of Rhode Island, Independence Square II, Kingston, RI 02881, USA; 2 Health Studies Program, University of Rhode Island, Independence Square II, Kingston, RI 02881, USA; steven_cohen@uri.edu (S.A.C.); mgreaney@uri.edu (M.L.G.); 3 Department of Nutrition and Food Sciences, University of Rhode Island, Fogarty Hall, Kingston, RI 02881, USA; ggreene@uri.edu

**Keywords:** weight perception, weight satisfaction, physical activity, diet quality

## Abstract

*Background*: It remain unclear that the association between weight status, weight perception, weight satisfaction and the clustering of physical activity (PA) and dietary behaviors in adolescents. *Method*: A cross-sectional analysis of National Health and Nutrition Examination Survey and the US Department of Agriculture’s Food Patterns Equivalents 2007–2014 data from adolescents aged 12–17 years (*n* = 2965) was conducted. Multivariable logistic regression models adjusted for demographic characteristics examined the association between weight status, weight perception, weight satisfaction, and the four created lifestyle groups (healthier behaviors, healthier diet only, physically active only, unhealthier behaviors). *Results*: Males with obesity were more likely to be in the healthier diet only group than males with a normal weight (OR = 1.90, 95% CI: 1.02, 3.52). Similar patterns were found in males who perceived themselves as being overweight or having obesity (OR = 2.09, 95% CI: 1.09, 3.99) and males with obesity who perceived their weight status accurately (OR = 2.33, 95% CI: 1.12, 4.88). Female respondents who were satisfied with their weight were 59% less likely to be in the healthier diet only group than healthier behaviors group compared with females who were weight dissatisfied (OR = 0.41, 95% CI: 0.23, 0.75). This pattern was not observed in males. *Conclusions*: Clustering PA and dietary behaviors were associated with weight status and weight perception for males but not females. Weight satisfaction was associated with clustered PA and dietary behaviors for females. These findings are important for obesity prevention policies and programs to better address adolescents’ obesity and reduce health disparities in this population.

## 1. Introduction

Currently 20.6% of US adolescents aged 12–19 have obesity [1]. This high prevalence of obesity and its associated short- and long-term health risks [1,2] make addressing obesity in this population a public health priority. Obesity among adolescents is often attributed to inadequate physical activity (PA) and a poor diet [3,4,5,6,7,8], and obesity prevention efforts often focus on increasing PA and promoting healthy diets to promote energy balance [7,8,9,10,11]. However, most available studies examining the relationships between lifestyle behaviors and obesity among adolescents focus on either PA or dietary behaviors [4,5,6,7,8]. Although an understanding of factors associated with both PA and dietary behaviors is needed to inform obesity prevention efforts for adolescents, research examining these behaviors simultaneously is lacking.

Weight perception, the way in which individuals view their body weight, may be associated with adolescents PA and dietary behaviors [12]. Some evidence suggests that adolescents with self-perceived overweight or obesity, regardless of weight perception accuracy, are less physically active than those who perceive themselves as being normal weight [6,13,14,15]. They also are more likely to make unhealthier dietary choices [15,16,17]. However, other studies have had inconsistent results [18,19]. For example, Wong & Leatherdale found that adolescents who perceived themselves as being overweight or having obesity are not necessarily physically inactive when compared with their normal weight counterparts [18], and Fredrickson and colleagues found that adolescents’ awareness of being overweight was not associated with their dietary choices [19].

Weight satisfaction, how one feels about his/her weight, also maybe associated with adolescents’ PA and dietary behaviors [20]. Research examining the relationship between weight satisfaction, PA and dietary behaviors among adolescents is limited and inconsistent [19,21,22]. Therefore, there is a need to examine this relationship among a nationally representative sample of US adolescents. Thus, the purpose of the present study was to examine the association between adolescents’ weight status, weight perception, weight satisfaction and the clustering of PA and dietary behaviors. Given that associations could differ by sex, we examined these relationships in males and females respectively.

## 2. Methods

The current study was a cross-sectional analysis of data from the National Health and Nutrition Examination Survey (NHANES) and the US Department of Agriculture’s Food Patterns Equivalents (USDA-FPE) 2007–2014 datasets. NHANES data were collected via questionnaires (e.g., demographics, weight perception or satisfaction, 24-h dietary recall) or measured (e.g., height and weight) with response rates vary from 70–80% [23,24]. The USDA-FPE data examined in this study were the dietary components assessed via 24-h dietary recalls [24,25]. The two datasets were merged using participants’ study identification numbers and days of 24-h recalled dietary data. The present study was exempted by University of Rhode Island Institutional Review Board as the data used for the study are de-identified.

### 2.1. Analytic Sample

A total of 40,617 respondents were abstracted from NHANES and the USDA-FPE 2007–2014 datasets. Of these respondents, 3920 were 12–17 years of age. Of these, 3041 had complete data for all examined variables including two days of dietary recalls. Of the 3041 respondents, 2.5% (*n* = 76) were underweight based on body mass index (BMI, <5th percentile for BMI), and were excluded due to possible underweight-related psychological or physical pathology issues [26] as well as the small sample size. The final analytic sample included 2965 adolescents.

### 2.2. Weight Status

BMI was calculated using height and weight that was measured at the Mobile Examination Center (MEC). BMI was used to determine weight status according to 2000 Centers for Disease Control and Prevention growth charts (0–20 years): obesity (≥95th percentile), overweight (≥85th percentile but <95th percentile), normal weight (≥5th percentile but normal <85th percentile, and underweight (<5 the percentile) [27].

### 2.3. Weight Perception and Weight Perception Accuracy

Respondents’ weight perception was measured by a single-item question that asked them how they considered their weight with the response options of fat or overweight, about right weight, and thin [23]. Weight perception accuracy (accurate, inaccurate) was determined by comparing perceived and measured weight status (normal, overweight, obese). If the two measures were concordant, weight perception accuracy was considered to be accurate and if the two measures were discordant weight perception accuracy was deemed inaccurate [6].

### 2.4. Weight Satisfaction

Weight satisfaction was measured one of two ways depending on respondents’ age. For respondents aged 12–15 years, it was assessed by a single item that asked what they would like to do about their weight with the response options of: (1) lose weight, (2) gain weight, (3) stay the same or (4) not trying to do anything about weight [23]. Respondents who wanted to “gain or lose weight” were classified as being weight dissatisfied while those who reported that they were trying to “stay the same or were not trying to do anything about their weight” were reported as being weight satisfied. Respondents aged 16–17 were asked whether they would like to change their weight with the response options of: (1) weigh more, (2) weigh less or (3) weigh the same [23], and those who reported that they would like to weigh more or less were classified as being weight dissatisfied while those who reported that they would like to weigh same were classified as being weight satisfied.

### 2.5. Physical Activity

PA was measured in three domains (work, travel, and recreational) using the Global Physical Activity Questionnaire [23]. The metabolic equivalent of task (MET) minutes of moderate to vigorous PA per week in each domain was determined and then summed to calculate total PA time per week [23,28]. Respondents who participated in ≥1680 MET-minutes of PA per week were classified as meeting current PA recommendations, all others were classified as not meeting PA recommendations [29].

### 2.6. Diet Quality

Diet quality was determined using the National Cancer Institute’s simple Healthy Eating Index 2015 (HEI-2015) scoring algorithm [30,31], which is a reliable and valid measure of diet quality [24,30]. The HEI-2015 uses information from two 24-h dietary recalls conducted by NHANES to assess 13 dietary components (e.g., saturated fats, whole grains, etc.) [30]. The 13 components were summed to create total diet quality scores (range 0 to 100) with a higher score indicating better diet quality and greater adherence to the 2015–2020 Dietary Guidelines for Americans [31]. Participants were stratified into tertiles based on their total dietary quality scores: (1) lower quality diet (scores <42.1); (2) intermediate quality diet (scores 42.1–51.7); (3) healthier diet (scores >51.7). The top tertile was viewed as being indicative of a healthier diet whereas another two tertiles (lower quality diet and intermediate quality diet) were considered to indicate eating a less healthful diet.

### 2.7. Lifestyle Groups

Respondents were classified into four lifestyle groups based on their PA and dietary behaviors [29,31]: Group 1, healthier behaviors: meeting PA recommendations and eating a healthier diet; Group 2, healthier diet only: eating a healthier diet but not meeting PA recommendations; Group 3, physically active only: meeting PA recommendations but eating a less healthful diet; and Group 4, unhealthier behaviors: not meeting PA recommendations and eating a less healthful diet.

### 2.8. Demographic Characteristics

Examined demographic characteristics included age, sex, race/ethnicity (non-Hispanic White, non-Hispanic Black, Mexican American, other Hispanic, others) and parental education levels (high school or less, college or above) [25]. Family income and family size was used to calculate the poverty-to-income ratio that was then used to determine if the household income was at/above (≥1) or below (<1) federal poverty level [32]. NHANES has sampling design to assure reliable estimates of various US population subgroups thus participants are representative of the US population [24,33].

### 2.9. Statistical Analysis

The MEC exam 2-year weights were used for all analyses [33]. Descriptive results were obtained for the sample and are presented as mean ± standard error for continuous variables and frequencies and proportions for categorical variables. For the sex-specific prevalence of weight status, weight perception, weight perception accuracy, and weight satisfaction by the lifestyle groups, *p*-values for continuous variables were obtained by performing PROC SURVEYREG, and *p*-values for categorical variable were obtained by performing PROC SURVEYLOGISTC to perform the adjusted analyses, adjusting for age, race/ethnicity, parental education levels, and whether respondents lived at/above or below the poverty line. Bonferroni corrections were conducted for all multiple comparisons.

To examine the association between weight status, weight perception, weight perception accuracy, and weight satisfaction (independent variables) and the four lifestyle groups (dependent variable), multivariable logistic regression models were performed with the use of PROC SURVEYLOGISTIC, with GLOGIT link (multinomial logistic model) with Group 1, healthier behaviors group, being the reference category to estimate the adjusted odds ratios. All models were adjusted for age, race/ethnicity, parental education level, and poverty status, and stratified by sex. All analyses were conducted using SAS version 9.4 (SAS Institute Inc., Cary, NC, USA) and significance was set at *p* < 0.05.

## 3. Results

Respondents’ mean age was 14.5 years, approximately half (49.7%) were females, 42.3% were racial/ethnic minorities, 39.6% had a parent with a high school education or less, 19.4% lived below the poverty line (see Table 1). About a quarter (20.8%) of respondents were classified as having obesity, 21.9% perceived themselves as being overweight or having obesity, 26% accurately perceived their weight as being overweight or obese, 55.8% were dissatisfied with their weight, 85.2% of respondents with obesity were dissatisfied with their weight.

In regards to the examined behaviors, 32.9% had diets that were classified as being a healthier diet and 54.9% met current PA recommendations. About one-fifth (18.6%) of respondents were classified as being in Group 1, the healthier behaviors group, indicating that they met current PA recommendations and ate a healthier diet (see Table 2). More females than males were in Group 2, healthier diet only group (17.2% females vs. 11.4% males, *p* < 0.001), and Group 4, unhealthier behaviors group (36.9% vs. 24.7%, *p* < 0.001), whereas more males were in Group 3, physically active only group (44.5% males vs. 27.9% females, *p* < 0.001) (see Table 2).

**Figure 1:**
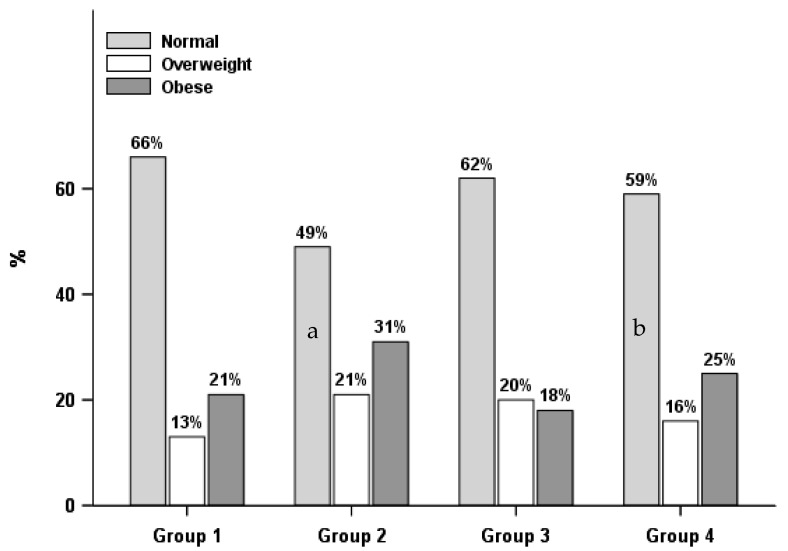
Lifestyle groups by weight status in males (*n* = 1504). Group 1: healthier behaviors; Group 2: healthier diet only; Group 3: physically active only; Group 4: unhealthier behaviors; a. Group 1 differed from Group 2 with a *p* < 0.05; b. Group 2 differed from Group 4 with a *p* < 0.05.

**Figure 2:**
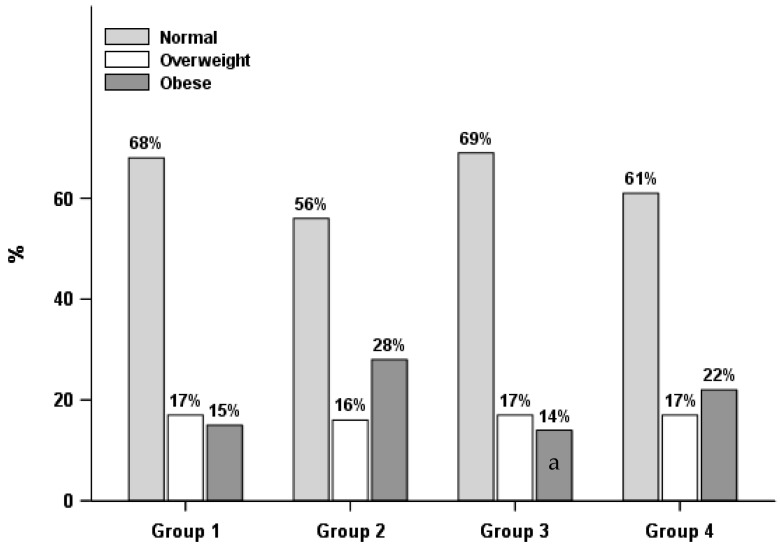
Lifestyle groups by weight status in females (*n* = 1461). Group 1: healthier behaviors; Group 2: healthier diet only; Group 3: physically active only; Group 4: unhealthier behaviors; a. Group 2 differed from Group 3 with a *p* < 0.05.

As shown in Table 3, Figure 1 and Figure 2, there were differences in weight status by lifestyle groups. A greater proportion of normal measured/perceived weight males as well as weight satisfied females were in Group 1, the healthier behaviors group, than Group 2, healthier diet only group, while males who perceived themselves as being overweight or having obesity and females who were weight dissatisfied were more likely to be in Group 2, the healthier diet only group, than Group 1, healthier behaviors group. Moreover, males with self-perceived overweight or obesity and females with obesity were more likely to be in Group 2, the healthier diet only group, than Group 3, the physically active only group.

Comparing Group 1, the healthier behaviors group, to the other three lifestyle groups by weight categories, males with overweight or obesity were more likely to be in Group 2, the healthier diet only group, than males with normal weight (see Table 4). More specifically, males with obesity were 90% more likely to be in Group 2, the healthier diet only group, than Group 1, healthier behaviors group, compared to males of normal weight (OR = 1.90, 95% CI: 1.02, 3.52). Similarly, males who perceived themselves as being overweight or having obesity (OR = 2.09, 95% CI: 1.09, 3.99) and those who accurately perceived themselves as having obesity (OR = 2.33, 95% CI: 1.12, 4.88) were more likely to be in Group 2 than Group 1. Males who accurately perceived themselves as being overweight were more likely to be in Group 2, the healthier diet only group (OR = 3.87, 95% CI: 1.01, 15.69) or Group 4, unhealthier behaviors group (OR = 3.15, 95% CI: 1.00, 10.04) than males who accurately perceived themselves as being normal weight (Table 4). No statistical difference was observed between males who were satisfied with their weight and those who were weight dissatisfied.

For females, there was no significant association between weight status, weight perception or weight perception accuracy by lifestyle groups. Females who were satisfied with their weight were 59% less likely to be in Group 2, healthier diet only group, than Group 1, the healthier behaviors group, compared with those who were dissatisfied with their weight (OR = 0.41, 95% CI: 0.23, 0.75).

## 4. Discussion

The present study used a nationally representative sample of adolescents to examine differences in four lifestyle groups characterized by the clustering of PA and dietary behaviors by weight status, weight perception, and weight satisfaction. Study results extended previous research findings by documenting that males with overweight or obesity or self-perceived overweight or obesity were less likely to meet PA recommendations than males with normal weight but diet quality did not differ [34]. Results of the current study also determined that females who were satisfied with their weight were more likely to meet PA recommendations and have a healthful diet than those who were dissatisfied with their weight. These findings indicate that lifestyle behaviors for males differ by weight status and weight perception whereas lifestyle behaviors for females differ by weight satisfaction.

In the present study, 54.9% of respondents were classified as meeting current PA recommendations, 32.9% had healthier diets. However, only 18.6% of the sample met PA recommendations and ate a more healthful diet. Although these finding are consistent with previous studies examining PA and/or diet quality among adolescents [4,11,35,36], no prior studies to our knowledge, examined the clustering of PA and dietary behaviors using a representative sample of US adolescents. Moreover, no identified studies assessed diet quality using the HEI-2015, which examines adherence to 2015–2020 dietary Guidelines for Americans [30]. The HEI-2015 differs from prior versions in assessed components and scoring, but, the average diet quality score of 47.5 in the current study is comparable to HEI-2010 mean score of 48.4 found in adolescents aged 12–18 from NHANES 2011–2012 [37]. Although the upper tertile (HEI-2015 scores >51.7) was defined as being a healthier diet for this study, according to the original HEI rating system, scores 51–80 indicate a diet that needs improvement [38]. Furthermore, the present study revealed sex differences across lifestyle groups: more females than males were in the healthier diet only group and the unhealthier behaviors group whereas more males than females were in the physically active only group. This finding is consistent with prior research that had found males to be more physically active than females [6,14] but differs from findings from other research who found that males had better dietary quality than females [35,39]. However, such results should be interpreted with caution because the clustering of PA and dietary behaviors were not examined in these studies [6,14,35,39].

Study findings indicate that males with overweight or obesity or who perceived themselves as being overweight or having obesity were more likely to consume a healthier diet but less likely to meet the PA recommendations than males with a normal weight or perceived their weight to be normal. However, similar results were not found among females. This finding differs from that of prior studies who have found that adolescents with self-perceived overweight or obesity were physically inactive [6,14,15] but not related to dietary choices [40], although comparisons between these studies should be made with caution considering that the current study examined individuals’ PA and dietary behavior cluster. Moreover, the present study found that males who accurately perceived themselves as being overweight were more likely have unhealthier lifestyle behaviors. These findings suggest that for males recognizing oneself as being overweight may be a barrier to participating in PA and eating a healthier diet but this might not the case for males with obesity. These findings also suggest that sex differences in the relationship of weight status, weight perception and lifestyle behaviors should be considered while tailoring suitable obesity prevention or intervention strategies to address the unique needs of males and females with obesity in different lifestyle behavior groups.

Additionally, although the percentage of adolescents who were weight satisfied in this study is comparable to other studies [19,40], no difference in weight satisfaction by sex were observed as other studies have found [19,40]. This could be due to the use of a single item assessing weight satisfaction in the current study compared to other research [19,40]. Furthermore, differences in weight satisfaction by lifestyle groups were observed only among females. Females who satisfied with their weight were more likely in the healthier behaviors group, meeting PA recommendations and eating a healthier diet, than females who dissatisfied with their weight. Fredrickson and colleagues also have found that weight satisfaction is associated with healthy behaviors such as meeting the daily fruit recommendations but not related to daily servings of vegetables or PA [19]. Nevertheless, the present study extends previous research by examining PA and overall diet quality in tandem, which provides important insights for obesity prevention efforts and support the idea that weight satisfaction may serve as motivating factor for females engaging in healthy behaviors.

Study strengths include the use of a nationally representative sample that is racially/ethnically diverse (42.3% identifying as racial/ethnic minorities) and parents with less than a college education (39.6%), to examine the association of weight status, weight perception, weight satisfaction, and the clustering of PA and dietary behaviors. An additional study strength is use of measured height and weight to determine weight status as well as the use of 24-h recall methodology instead of a food frequency questionnaire. This also is the first study using HEI-2015 as an overall measure of dietary quality in adolescents. Study limitations include its cross-sectional design which does not allow for causality evaluation, weight satisfaction assessed using a single-item, and PA measured using a self-reported instrument. In addition, healthier PA was defined as meeting guidelines but since no guidelines exist for HEI-2015, healthier was defined as the upper tertile. This resulted in a greater proportion of adolescents meeting the PA cutoff for healthier than the dietary cutoff for healthier, thus results must be interpreted with caution. Nevertheless, all the measures are widely used and the study utilized validated instruments [24,28,29,30].

## 5. Conclusions

There were differences in weight status and weight perception by lifestyle group, and these differences were largely driven by PA for males and diet quality for females. Males with overweight or obesity were more likely not to meet PA recommendations but tended to have a higher quality diet than males with normal weight. Similar pattern was also observed in males with self-perceived overweight or obesity. Whereas females who were weight satisfied were more likely to meet the PA recommendation and eat a healthier diet than females who were dissatisfied with their weight. These results indicate that the importance of weight status, weight perception, and weight satisfaction in the adoption of healthier lifestyle behaviors particularly for individuals with overweight or obesity. Our findings also suggest that examining PA and dietary behavior cluster instead of either/or in the present study provides important insights on lifestyle behaviors difference that can be used to frame future research or interventions.

## Figures and Tables

**Table 1:** Sample characteristics stratified by sex (*n* = 2965).

	Total	Males	Females	*p* Value
	*n* = 2965	*n* = 1504	*n* = 1461	
Gender, *n* (weighted %)				
Males	1504 (50.3)	-	-	-
Females	1461 (49.7)	-	-	-
Age (years) (mean ± SE)	14.5 ± 0.0	14.5 ± 0.1	14.6 ± 0.1	0.409
Ethnicity, *n* (weighted %)				
Non-Hispanic White	827 (57.7)	435 (57.4)	392 (57.9)	0.804
Non-Hispanic Black	748 (14.1)	378 (14.0)	370 (14.2)	0.856
Mexican American	695 (13.7)	346 (13.8)	349 (13.5)	0.787
Other Hispanic	342 (6.5)	171 (6.6)	171 (6.4)	0.772
Others	353 (8.0)	174 (8.1)	179 (7.9)	0.857
Parent education level, *n* (weighted %)				
High school or less	1420 (39.6)	719 (38.9)	701 (40.3)	0.524
College or above	1451 (60.4)	736 (61.1)	715 (59.7)	0.524
Poverty to income ratio, *n* (weighted %)				
<1.0	793 (19.4)	394 (19.0)	399 (19.9)	0.589
≥1.0	1941 (80.6)	994 (81.0)	947 (80.1)	0.589
BMI (kg/m^2^)	23.5 ± 0.1	23.4 ± 0.2	23.6 ± 0.2	0.446
Weight status, *n* (weighted %)				
Normal weight	1765 (62.1)	905 (60.6)	860 (63.6)	0.308
Overweight	521 (17.1)	254 (17.6)	267 (16.6)	0.604
Obesity	679 (20.8)	345 (21.8)	334 (19.8)	0.291
Perceived weight status, *n* (weighted %)				
Thin	196 (5.5)	128 (7.3)	68 (3.6)	<0.001 *
Normal	2055 (72.6)	1093 (75.6)	962 (69.6)	<0.001 *
Overweight/Obese	714 (21.9)	283 (17.1)	431 (26.8)	<0.001 *
Accurate weight perception ^#^				
Normal	1481 (74.1)	760 (76.3)	721 (72.0)	0.072
Overweight	148 (6.4)	46 (4.3)	102 (8.3)	0.012 *
Obese	471 (19.6)	217 (19.4)	254 (19.7)	0.898
Weight satisfaction, *n* (weighted %)				
Satisfied	938 (44.2)	471 (46.0)	467 (42.6)	0.191
Dissatisfied	1358 (55.8)	634 (54.0)	724 (57.4)	0.191
Weight satisfaction by weight category, *n* (weighted %)				
Normal weight				
Satisfied	727(61.6)	343(61.6)	384(61.6)	0.993
Dissatisfied	474(38.4)	225(38.4)	249(38.4)	0.993
Overweight				
Satisfied	134(33.8)	80(45.0)	54(21.9)	<0.001 *
Dissatisfied	313(66.2)	132(55.0)	181(78.1)	<0.001 *
Obesity				
Satisfied	77(14.8)	48(18.7)	29(10.6)	0.079
Dissatisfied	571(85.2)	277(81.3)	294(89.4)	0.079
Physical activity (PA)				
Total PA (MET-minutes/week)	2798.1 ± 85.2	3350.7 ± 121.7	2239.8 ± 92.1	<0.001 *
Met PA recommendation ^$^, *n* (weighted %)	1491 (54.9)	900 (63.8)	591 (45.9)	<0.001 *
Sedentary (sitting) in minutes/day	498.1 ± 5.5	489.1 ± 7.1	507.1 ± 6.4	0.026 *
Diet quality score (HEI 2015)				
Total diet quality (mean ± SE)	47.5 ± 0.3	46.9 ± 0.4	48.1 ± 0.4	0.016 *
Tertile classification of total diet quality score, *n* (weighted %)				
First tertile (<42.1)	978 (34.3)	517 (34.7)	461 (34.0)	0.741
Second tertile (42.1–51.7)	978 (32.7)	518 (34.6)	460 (30.8)	0.108
Third tertile (>51.7)	1009 (32.9)	469 (30.7)	540 (35.2)	0.023 *

Note: Data are present as weighted mean ± standard error (SE) unless otherwise specified; ^$^ accumulated ≥1680 MET minutes/week for 12–17 years old, HEI = Health Eating Index; ^#^ weigh perception accuracy defined as consistency of perceived and measured weight status. If the two measures were concordant, weight perception accuracy was considered to be accurate; * *p* < 0.05.

**Table 2:** Lifestyle groups stratified by sex (*n* = 2965).

	Total	Males	Females	Proportion Difference between Females and Males (95% CI)	*p* Value
Groups, *n* (weighted %)	*n* = 2965	*n* = 1504	*n* = 1461	*n* = 2965	
Group 1: Healthier behaviors	515 (18.6)	282 (19.3)	233 (18.0)	−1.27 (−4.84, 2.31)	0.481
Group 2: Healthier diet only	494 (14.3)	187 (11.4)	307 (17.2)	5.75 (2.56, 8.94)	<0.001 *
Group 3: Physically active only	976 (36.3)	618 (44.5)	358 (27.9)	−16.63 (−20.1, −13.15)	<0.001 *
Group 4: Unhealthier behaviors	980 (30.8)	417 (24.7)	563 (36.9)	12.14 (8.48, 15.81)	<0.001 *

Note: Data are present as weighted mean ± standard error unless otherwise specified; CI = confidence interval; * *p* < 0.05.

**Table 3:** Associations between weight perception, weight satisfaction and lifestyle groups (*n* = 2965).

	Group 1: Healthier Behaviors	Group 2: Healthier Diet Only	Group 3: Physically Active Only	Group 4: Unhealthier Behaviors	Overall *p* Value
**Males (*n* = 1504)**	***n* = 282 (19.3%)**	***n* = 187 (11.4%)**	***n* = 618 (44.5%)**	***n* = 417 (24.7%)**	
Weight status					
Normal weight	173 (65.9)	92 (48.7) ^a^	386 (62.1)	254 (59.2) ^c^	0.017 *
Overweight	43 (12.8)	37 (20.6)	109 (20.1)	65 (15.6)	0.041 *
Obese	66 (21.4)	58 (30.7)	123 (17.7)	98 (25.2)	0.038 *
Perceived weight status					
Thin	18 (5.0)	16 (7.8)	58 (7.9)	36 (7.9)	0.258
Normal	210 (78.1)	121 (66.4) ^a^	466 (79.3)	296 (71.0)	0.022 *
Overweight/Obese	54 (16.9)	50 (25.8) ^a^	94 (12.8) ^b^	85 (21.1)	0.005 *
Accurate weight perception					
Normal	150 (79.3)	73 (61.7) ^a^	323 (81.5) ^b^	214 (71.4)	0.007 *
Overweight	7 (3.2)	9 (6.9)	13 (2.5)	17 (6.9)	0.048 *
Obese	41 (17.5)	38 (31.5)	75 (16.0)	63 (21.7)	0.045 *
Weight satisfaction					
Satisfied	94 (53.0)	54 (43.8)	193 (44.7)	130 (43.9)	0.468
Dissatisfied	119 (47.0)	94 (56.2)	255 (55.3)	166 (56.1)	0.468
**Females (*n* = 1461)**	***n* = 233 (18.0%)**	***n* = 307 (17.2%)**	***n* = 358 (27.9%)**	***n* = 563 (36.9%)**	
Weight status					
Normal weight	145 (68.0)	165 (56.3)	216 (68.8)	334 (60.9)	0.222
Overweight	42 (16.7)	58 (15.7)	74 (17.0)	93 (16.6)	0.956
Obese	46 (15.3)	84 (28.1)	68 (14.2) ^b^	136 (22.5)	0.007 *
Perceived weight status					
Thin	9 (3.4)	16 (4.7)	15 (2.8)	28 (3.8)	0.538
Normal	168 (73.7)	181 (59.2)	250 (75.4) ^b^	363 (68.1)	0.023 *
Overweight/Obese	56 (23.0)	110 (36.1)	93 (21.8) ^b^	172 (28.1)	0.049 *
Accurate weight perception					
Normal	132 (76.4)	132 (60.6)	182 (78.7) ^b^	275 (69.7)	0.036 *
Overweight	15 (8.2)	30 (11.1)	25 (7.8)	32 (7.4)	0.863
Obese	35 (15.5)	63 (28.3)	49 (13.5)	107 (22.9)	0.011 *
Weight satisfaction					
Satisfied	88 (52.0)	80 (29.1) ^a^	110 (43.5)	189 (43.8) ^c^	0.009 *
Dissatisfied	103 (48.0)	175 (70.9) ^a^	178 (56.5)	268 (56.2) ^c^	0.009 *

Note: Data are presented as *n* (weighted %); *p*-value for continuous variables was obtained by performing PROC SURVEYREG, and *p*-value for category variable was obtained by performing PROC SURVEYLOGISTC to perform the adjusted analyses, adjusted for age, race, parental education level and poverty status; Bonferroni corrections were used for all multiple comparisons; ^a^ Group 1 differed from Group 2 with a *p* < 0.05; ^b^ Group 2 differed from Group 3 with a *p* < 0.05; ^c^ Group 2 differed from Group 4 with a *p* < 0.05; * *p* < 0.05.

**Table 4:** Adjusted odd ratios of lifestyle groups related to weight status, weight perception, and weight satisfaction (*n* = 2965).

	Group 2: Healthier Diet Only vs. Group 1: Healthier Behaviors		Group 3: Physically Active Only vs. Group 1: Healthier Behaviors		Group 4: Unhealthier Behaviors vs. Group 1: Healthier Behaviors	
**Males (*n* = 1504)**	**Adjusted ORs (95% CI)**	***p* Value**	**Adjusted ORs (95% CI)**	***p* Value**	**Adjusted ORs (95% CI)**	***p* Value**
Weight status						
Normal weight	1.0 (REF)		1.0 (REF)		1.0 (REF)	
Overweight	2.33 (1.15, 4.68)	0.016*	1.73 (1.00, 3.00)	0.046 *	1.21 (0.67, 2.19)	0.528
Obese	1.90 (1.02, 3.52)	0.038*	0.85 (0.50, 1.44)	0.537	1.14 (0.69, 1.86)	0.605
Perceived weight status						
Normal	1.0 (REF)		1.0 (REF)		1.0 (REF)	
Thin	0.89 (0.34, 2.32)	0.805	1.43 (0.71, 2.88)	0.308	1.45 (0.71, 2.95)	0.301
Overweight/Obese	2.09 (1.09, 3.99)	0.024*	0.82 (0.49, 1.38)	0.444	1.37 (0.87, 2.16)	0.164
Accurate weight perception						
Normal	1.0 (REF)		1.0 (REF)		1.0 (REF)	
Overweight	3.87 (1.01, 15.69)	0.047 *	1.09 (0.33, 3.63)	0.886	3.15 (1.00, 10.04)	0.048 *
Obese	2.33 (1.12, 4.88)	0.022 *	0.90 (0.48, 1.68)	0.74	1.18 (0.66, 2.10)	0.571
Weight satisfaction						
Dissatisfied	1.0 (REF)		1.0 (REF)		1.0 (REF)	
Satisfied	0.69 (0.40, 1.19)	0.174	0.73 (0.46, 1.15)	0.165	0.75 (0.45, 1.24)	0.256
**Females (*n* = 1461)**						
Weight status						
Normal weight	1.0 (REF)		1.0 (REF)		1.0 (REF)	
Overweight	1.05 (0.51, 2.17)	0.885	1.02 (0.51, 2.05)	0.953	1.06 (0.56, 2.00)	0.856
Obese	1.97 (0.96, 4.04)	0.058	0.92 (0.51, 1.66)	0.779	1.52 (0.85, 2.73)	0.153
Perceived weight status						
Normal	1.0 (REF)		1.0 (REF)		1.0 (REF)	
Thin	1.47 (0.37, 5.80)	0.576	0.54 (0.13, 2.19)	0.383	1.04 (0.27, 3.98)	0.949
Overweight/Obese	1.53 (0.83, 2.84)	0.167	0.84 (0.51, 1.38)	0.472	1.25 (0.75, 2.10)	0.378
Accurate weight perception						
Normal	1.0 (REF)		1.0 (REF)		1.0 (REF)	
Overweight	1.41 (0.48, 4.20)	0.525	0.86 (0.39, 1.87)	0.69	0.94 (0.37, 2.36)	0.889
Obese	1.80 (0.87, 3.72)	0.105	0.79 (0.41, 1.53)	0.477	1.42 (0.74, 2.70)	0.28
Weight satisfaction						
Dissatisfied	1.0 (REF)		1.0 (REF)		1.0 (REF)	
Satisfied	0.41 (0.23, 0.75)	0.003 *	0.74 (0.43, 1.27)	0.269	0.74 (0.41, 1.34)	0.307

Note: ORs = odds ratios; REF = reference; Adjusted ORs were obtained by PROC SURVEYLOGISTIC using the generalized logit model with the LINKG = LOGIT option (multinomial logistic model), adjusted for age, race/ethnicity, parental education level, poverty status; * *p* < 0.05.

## Declaration

All data are publicly accessible and available in the NHANES repository, https://wwwn.cdc.gov/nchs/nhanes/sasviewer.aspx or United States Department of Agriculture’s Food Patterns Equivalents Database, https://www.ars.usda.gov/northeast-area/beltsville-md-bhnrc/beltsville-human-nutrition-research-center/food-surveys-research-group/docs/fped-data-tables/.
